# In Vivo Confocal Microscopy and Anterior Segment Optical Coherence Tomography Analysis of the Microcystic Keratitis

**DOI:** 10.1155/2020/8871904

**Published:** 2020-12-17

**Authors:** Michał Dembski, Anna Nowińska, Klaudia Ulfik, Sławomir Teper, Edward Wylęgała

**Affiliations:** Faculty of Medical Sciences in Zabrze, Medical University of Silesia, Chair and Department of Ophthalmology, Panewnicka 65, Katowice 40-760, Poland

## Abstract

**Purpose:**

To describe the findings of in vivo confocal microscopy (IVCM) and anterior segment optical coherence tomography (AS-OCT) in a case of bilateral acute microcystic epitheliopathy after daily soft contact lens wear.

**Methods:**

IVCM and AS-OCT were used in the course of the bilateral epitheliopathy of a 23-year-old patient at the acute stage of the disease and at recovery after four days of treatment. The images were analyzed and compared.

**Results:**

On AS-OCT of the right eye, general hyperreflectivity and the increased thickness of the central corneal epithelium to 150 *μ*m with numerous hyporeflective small, oval cysts were revealed and resolved completely at day 4 after diagnosis and treatment. AS-OCT scans of the left eye revealed oval shaped, hyperreflective material within the corneal epithelium. IVCM of both eyes showed numerous microcysts of different sizes filled with hyperreflective material mostly within superficial epithelial layers. Epithelial cells showed a polymorphism along with disruption of a cytoarchitecture. Basal epithelial cells and anterior stroma showed inflammatory changes. Posterior corneal stroma and endothelium presented normal morphology.

**Conclusions:**

Microcystic keratitis appeared as localized microcystic inflammation of epithelium on AS-OCT, which was confirmed by IVCM. Both IVCM and AS-OCT are helpful diagnostic instruments in case of cystic inflammation of corneal epithelium.

## 1. Introduction

Keratitis is a disease characterized by the occurrence of inflammatory infiltration within one or several layers of the cornea such as the epithelium, Bowman layer, corneal stroma, and endothelium with the Descemet membrane. In most cases, keratitis is considered as an acute ophthalmologic condition and requires urgent treatment [1].

There are two main types of keratitis: infectious and noninfectious. Infectious keratitis includes bacterial, viral, fungal, and protozoal inflammations. Noninfectious keratitis contains many diseases, caused by an abnormal immunological reaction or disturbed physiological processes of the eye's surface [2].

Differential diagnosis of keratitis is a major challenge due to the frequent occurrence of similar clinical symptoms in both infectious and noninfectious inflammations. Nonspecific moderate or severe pain, decreased visual acuity, photophobia, and eye surface irritation are subjective symptoms reported by patients with keratitis. The diagnosis is mainly based on physician's clinical experience in addition to microbiological analysis results. The positive predictive value of physician's clinical experience had been shown to be very variable depending on the etiology of the infection. The rate of positive culture in cases of infectious keratitis has varied widely between 40 and 70% due to a variety of limiting factors [3–5]. For these reasons, delays in diagnosis and treatment are common, and there is a strong necessity to utilize new, reliable modalities in the diagnostic process of keratitis. Both optical coherence tomography and confocal microscopy are noninvasive imaging modalities, which were already proved to help in differential diagnosis, especially in cases of atypical or complex infectious keratitis [6–9].

The main risk factors which should be considered while diagnosing corneal inflammation include using of contact lenses, corneal injury with epithelial discontinuity, viral diseases, and some systemic diseases such as autoimmune diseases.

Up to 80% of corneal inflammations are found in contact lens wearers [10]. Prevalence of corneal inflammations in people wearing contact lenses is relatively constant over recent years and starts with 2–4 cases for 10,000 users per year in soft contact lens wearers and reaches up to 20 cases for 10,000 users per year in overnight contact lens wearers [11].

Microcystic keratitis is one of the subtypes of keratitis characterized by the appearance of small (diameter from 15 to 50 micrometers), irregular, and translucent inclusions in the corneal epithelium.

The causes of the occurrence of microcysts are not fully understood. Chronic hypoxia, inflammatory mechanisms, and mechanical pressure on corneal surface are considered as possible.

Microcystic keratitis is relatively common among people wearing contact lenses. Differential diagnosis with other infectious and aseptic inflammations is required [12].

## 2. Case Report

A 28-year-old female patient was admitted to an eye emergency unit due to sudden, significant, and bilateral loss of visual acuity. No pain or photophobia was reported by the patient. Blurred and hazed vision was discovered by the patient after waking up in the morning.

Anamnesis revealed bilateral conjunctivitis, which occurred about a month ago and completely resolved within a week after treatment with topical tobramycin. Furthermore, the patient has been wearing corrective contact lenses (−3, 0 Dsph. both eyes) for about five years. Single use and daily wear soft contact lenses containing 31% nelfilcon A and 69% water were used by the patient. At admission, BCVA was RE 0, 6 and LE 0, 4. Intraocular pressure was RE 14 mmHg and LE 12 mmHg.

**Figure 1:**
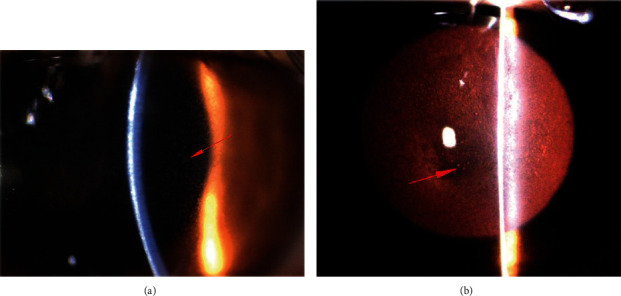
Photography of the anterior segment of the eye. (a) Numerous microcysts visible in the epithelial layer (red arrow) (mag. 16x). (b) Epithelial microcysts visible in retroillumination (red arrow) (mag. 10x).

Slit-lamp examination showed numerous small inclusions in the superficial and central cornea without corneal erosion and without corneal haze. Less severe changes were found in the peripheral part of the cornea. There was an asymmetry of corneal abnormalities between eyes, with the right eye revealing more severe and advanced corneal changes. Fluorescein staining was negative in both eyes. No signs of acute inflammation were discovered during examination. The other structures of the eye were normal (Figures 1(a) and 1(b)).

**Figure 2:**
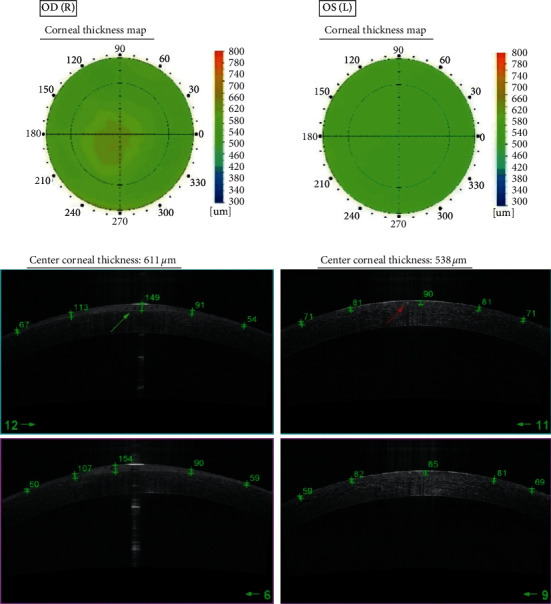
Comparison of representative SS OCT scans in the right and left eye. OD.SS OCT pachymetry map showing the increase of CCT to 611 *μ*m. High-resolution SS OCT cornea scan showing numerous hyporeflective small and oval cysts (green arrow) as well as increased thickness of the central corneal epithelium to 154 *μ*m. OS.SS OCT pachymetry map showing the CCT of 538 *μ*m. High-resolution SS OCT cornea scan showing few oval shaped, hyperreflective material within the central corneal epithelium (red arrow). Central epithelium thickness is increased to 90 *μ*m.

Optical coherence tomography of the anterior segment of the eye (DRI Triton, Topcon, Tokyo, Japan) revealed changes of varying severity between eyes. Both corneas were oedematous with the increase of the central corneal thickness to 611 *μ*m of the right eye and to 538 *μ*m of the left eye. The increase of the corneal thickness was due to epithelial oedema mainly in the central part of the cornea which was demonstrated on the corneal map. Peripheral epithelium thickness of both eyes ranged from 54 to 71 *μ*m. On AS-OCT of the right eye, general hyperreflectivity and the increased thickness of the central corneal epithelium to 150 *μ*m with numerous hyporeflective small, oval cysts were revealed. The hyporeflective cysts were located within 2 mm of the central corneal part. AS-OCT scans of the left eye revealed few oval-shaped, hyperreflective material within the central corneal epithelium. Reflectivity of the corneal epithelium was normal and the increase of the central thickness was upto 90 *μ*m which is significantly less severe compared to the right eye (Figure 2).

**Figure 3:**
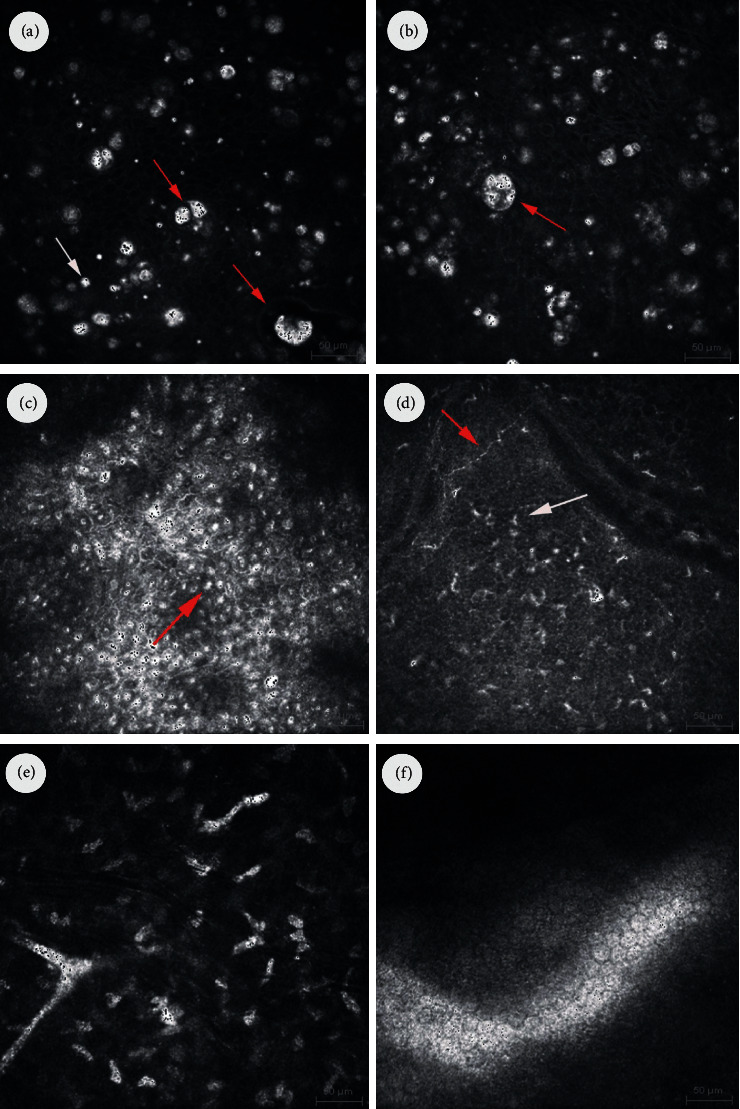
Confocal micrographs of the cornea. (a) IVCM micrograph of the superficial epithelium showing numerous small microcysts filled with highly reflective homogenous material (white arrow). Larger cysts filled with heterogenous hyporeflective and hyperreflective material (red arrows). (b) IVCM micrograph of the epithelium showing polymorphism along with disruption of a cytoarchitecture and disruption of the layered structure of the epithelium. Large cysts filled with heterogenous hyporeflective and hyperreflective material (red arrows). (c) IVCM image at the level of basal cells of corneal epithelium showing inflammatory changes. Brightly reflective and round structures visible at the centre of basal epithelial cells (red arrow). (d) IVCM micrograph of the Bowman layer showing increased density of Langerhans cells (white arrow) and activated keratocytes in the anterior stroma. Corneal nerve with normal morphology (red arrow). (e) IVCM image at the level of intermediate stroma showing activated keratocytes. (f) IVCM image of the endothelium with normal structure.

In vivo confocal microscopy (HRT 3, Heidelberg Engineering GmbH, Germany) of both eyes showed similar morphology changes, but the intensity was also significantly higher in the right eye. There were numerous microcysts with the size ranged from 8 *μ*m to 38 *μ*m. Majority of the small microcysts were filled with highly reflective homogenous material, but larger cysts were filled with heterogenous hyporeflective and hyperreflective material. Epithelial cells showed a polymorphism along with disruption of a cytoarchitecture and disruption of the layered structure of the epithelium. Basal epithelial cells and anterior stroma showed inflammatory changes. There were brightly reflective and round structures at the centre of basal epithelial cells, increased density of Langerhans cells, and activated keratocytes in the anterior stroma. Corneal nerves within the Bowman layer were present, but the visibility of the layer was disrupted by optical shadows from the cysts, hyperreflective structures at the centre of basal cells, and increased density of the Langerhans cells. Posterior corneal stroma and endothelium presented normal morphology (Figure 3).

The patient also underwent basic laboratory analysis including morphology with a microscopic smear, C-reactive protein, Bence–Jones protein in the urine, and a proteinogram. All results were normal.

Acute microcystic keratitis was diagnosed based on slit-lamp examination and AS-OCT and IVCM results.

The patient was treated with topical 0.1% dexamethasone in both eyes 7 times a day along with lubricating eye drops without preservatives.

After four days of treatment, a significant remission of symptoms was observed. At the follow-up visit on the fourth day of the treatment, BCVA was RE 1, 0 and LE 1, 0. Slit lamp examination along with OCT and IVCM were performed.

OCT exam revealed the complete remission of symptoms, and both central corneal thickness and central epithelial thickness returned to normal and were RE 552 *μ*m, 54 *μ*m and 547 *μ*m, and 56 *μ*m, respectively. Also, there was no disruption of the epithelium reflectivity. IVCM exam did not show any cysts within the superficial layers of the epithelium, but there were still signs of the inflammation activation of the basal epithelial cells and anterior corneal stroma, but the intensity was minimal compared to the analysis performed at diagnosis.

The patient was advised to use topical dexamethasone once per day for both eyes accompanied with artificial tears. There were no recurrences reported at the next follow-up visits.

## 3. Discussion

To the best of our knowledge, this is the first report in the literature describing the usage of optical coherent tomography of the anterior segment of the eye and confocal microscopy in acute microcystic keratitis.

Corneal epithelial microcysts were first described in the 1970s in patients wearing hydrogel contact lenses. Depending on the study, the incidence of microcysts in the corneal epithelium was 4 percent in the first days of wearing the lenses and increased by up to 30% after 3 months of wearing the lenses. Some of the papers reported a 100 percent incidence of cysts in patients who wore contact lenses for six months and exceeded their approved daily use period. Their occurrence was correlated with the wearing time of the contact lenses and their oxygen permeability (Dk/L). Potential causes included delayed epithelial response to chronic corneal hypoxia and chronic mechanical compression on the epithelium. Visual acuity was not deteriorated by occurrence of microcysts. The time taken for cysts to disappear since stopping wearing the lenses was about 3 months [12].

Microcystic keratitis is a nonspecific inflammation. Corneal hypoxia due to the contact lens use, inflammation of the protozoan etiology, and corneal dystrophy such as Messman's dystrophy, Cogan's dystrophy, bullous keratopathy, and recurrent corneal erosion syndrome are possible causes [13].

In order to exclude protozoal keratitis, confocal microscopy was performed, which is a sensitive test excluding early stages of this inflammation [14]. Its specificity depends on the type of confocal structure visible in the exam and reaches 94% in the case of cysts and trophozoites, while much lower specificity of 50% is obtained when structures with “bright spot” morphology are visible [15]. In our case, any of the structures characteristic for protozoan inflammatory were not found in the IVCM. Signs of dystrophy or corneal endothelial damage were also not found. We have also excluded infectious anamnesis or exposure to chemicals as well as recurrent corneal erosions.

Irregular increase in the thickness of the central corneal epithelium was a characteristic feature observed in the OCT. Normal, mean thickness of the corneal epithelium in the population is 53.4 ± 4.6 *μ*m [16]. Nishino et al. performed both optical coherent tomography as well as confocal microscopy in vivo in patients with genetically confirmed Messman dystrophy (MECD: mutation in the KRT12 gene), which also should be taken into differential diagnosis in our patient. In case of MECD, the increase of the thickness of the corneal epithelium to 64.8 *μ*m was seen. Epithelial thickness remained constant throughout the cornea. In our case, however, the thickness of the corneal epithelium was larger and reached 100 um. Also, the largest epithelial thickness occurred in the central part of the cornea while the peripheral epithelium remained normal. Epithelial edema contributed to an increase in the CCT to about 611 microns, while Nishino et al. noted a reduction in the CCT to about 499 microns despite epithelial edema. In the case of Messman's dystrophy, the IVCM study highlights the smaller accumulation of hyperreflective material in the epithelial cell layer, changes in subepithelial nerve morphology, Bowman's membrane atrophy, and observable changes in the corneal stroma (punctate and needlelike hyperreflective deposits). Changes in IVCM in cystic keratopathy only affect the epithelial layer. Numerous, fine endothelial cysts that are gently stained with fluorescein show both similar signs for Messman dystrophy and microcystic keratitis. [17].

Hyperreflective dots limited to the epithelial layers of the cornea were also found in AS-OCT by Thanathanee et al. in all patients with microsporidial keratoconjunctivitis. There was no extension into the stromal layer in all cases. Moreover, the lesions were slightly raised above the epithelial surface in almost all cases (12/13). The first two findings are similar with observations visible in OCT in our patient. However, in our case, hyperreflective epithelial lesions did not elevate above the surface of the cornea. Another difference between our case and microsporidal infection is the red eye revealed in slit-lamp examination, which was presented in the cited paper [18].

Visual acuity is deteriorated in microcystic keratitis. The literature describes cases of VA large deteriorated (1/60) to slightly reduced (6/12). The symmetry of changes is noteworthy [19]. In our patient, however, visual acuity at admission varied significantly between the right and left eye. The mechanism of this phenomenon remains unexplained. Lenses' wear time, type, manufacturer, and type of storage fluid were the same.

Thinning of the central cornea is a possible outcome of microcystic keratitis [19]. In our case, the complication did not occur and CCT after recovery was 552 and 547 micrometres, respectively, for the right and left eye.

After excluding other causes of inflammation, we believe that the main cause of acute cystic response in our patient was hypoxia of the corneal epithelial cells. This potential reason was also mentioned by Tuft et al. in the article describing similar cases [19].

There are also unknown reasons why, despite many years of asymptomatic history of wearing contact lenses, the acute phase of the disease has occurred in our patient. It is possible that inflammatory mechanisms are involved in releasing the mechanisms of acute hypoxia. The corneal epithelium is constantly renewed by new cells produced by mitosis in the epithelial basement membrane and washing dead cells from the surface of the cornea by a tear film. The normal epithelium consists mainly of live cells, while a small part of them are dead cells. They do not differ morphologically from living cells, but they can be removed very simply because they do not form permanent connections with neighboring cells [20]. Hypoxia is a well-studied factor in causing premature apoptosis and cell necrosis [21]. It is possible, therefore, that the production of microcysts is caused by prematurely induced apoptosis among corneal epithelial cells, which did not manage to migrate to its surface. The accumulation of apoptosis products in the intercellular spaces of the epithelium seems to be confirmed by the IVCM study showing hyperreflective material in the epithelial spaces, as well as edema of some cells.

To conclude, both optical coherent tomography of the anterior segment of the eye and in vivo confocal microscopy are high utility instruments in the differential diagnosis of microcystic keratitis. OCT allowed to asses corneal thickness change dynamics, as well as a morphology assessment of the corneal epithelium and stroma. The exam determined the difference between central and peripheral epithelium. Confocal microscopy analysis revealed cellular structure changes, mainly epithelium showing polymorphism along with disruption of a cytoarchitecture and large cyst filled with heterogenous hyporeflective and hyperreflective material. The analysis allowed differential diagnosis with infectious keratitis, especially of protozoal and fungal etiology. Acute microcystic keratitis is a rare complication of using soft contact lenses due to swelling of hypoxic epithelial cells and the formation of intercellular cysts containing apoptosis products. Differential diagnosis of keratitis in patients wearing contact lenses should include microcystic inflammation despite its rare incidence.
